# Prematurity, Subclinical Intraamniotic Infection, and Fetal Biophysical Parameters: Is There a Correlation?

**DOI:** 10.1155/S1064744993000183

**Published:** 1993

**Authors:** Susan M. Cox, Scott Roberts, Percilis Roussis, Berry A. Campbell, Thomas E. Curry

**Affiliations:** 1 Department of Obstetrics and Gynecology University of Kentucky Lexington KY USA; 2 University of Texas Southwestern Medical Center, Dept. of OB/GYN, 5323 Harry Hines Boulevard Dallas TX 75235-9032 USA

## Abstract

*Objective:* This prospective study was undertaken to examine the effects of subclinical intraamniotic infection on fetal behavioral patterns.

*Methods:* Amniotic fluid was obtained from four groups of patients (n = 99): group 1, patients with preterm premature rupture of the fetal membranes (PPROM) without infection; group 2, patients with PPROM and infection; group 3, patients with preterm labor (PTL) and without infection; and group 4, patients with PTL and infection. Fetal biophysical profiles were obtained on admission to the labor suite. Amniotic fluid was analyzed for the presence of microorganisms and endotoxin to confirm intraamniotic infection; cytokines interleukin (IL)-1β, IL-6, and IL-8 were also assayed.

*Results:* We found no association between low scores for biophysical parameters and subclinical infection in patients with PPROM or PTL.

*Conclusions:* We could not demonstrate that upon a patient's admission to the labor hall absent fetal breathing and absent fetal movement, as well as reactivity, correlate with subclinical intraamniotic infection. Elevated cytokines, i.e. IL-1β, IL-6, and IL-8 were associated with subclinical chorioamnionitis.


Infectious Diseases in Obstetrics and Gynecology 1:76–81 (1993)
© 1993 Wiley-Liss, Inc.

### Prematurity, Subclinical Intraamniotic Infection, and Fetal Biophysical Parameters: Is There a Correlation?

**Susan M. Cox, Scott Roberts, Percilis Roussis, Berry A. Campbell, and Thomas E. Curry, Jr.**

*Department of Obstetrics and Gynecology, University of Kentucky, Lexington, KY*

ABSTRACT

***Objective:*** **This prospective study was undertaken to examine the effects of subclinical intraamniotic infection on fetal behavioral patterns.**

***Methods:*** **Amniotic fluid was obtained from four groups of patients (n = 99): group 1, patients with preterm premature rupture of the fetal membranes (PPROM) without infection; group 2, patients with PPROM and infection; group 3, patients with preterm labor (PTL) and without infection; and group 4, patients with PTL and infection. Fetal biophysical profiles were obtained on admission to the labor suite. Amniotic fluid was analyzed for the presence of microorganisms and endotoxin to confirm intraamniotic infection; cytokines interleukin (IL)-1β, IL-6, and IL-8 were also assayed.**

***Results:*** **We found no association between low scores for biophysical parameters and subclinical infection in patients with PPROM or PTL.**

***Conclusions:*** **We could not demonstrate that upon a patient's admission to the labor hall absent fetal breathing and absent fetal movement, as well as reactivity, correlate with subclinical intraamniotic infection. Elevated cytokines, i.e. IL-1β, IL-6, and IL-8 were associated with subclinical chorioamnionitis.** © 1993 Wiley-Liss, Inc.

KEY WORDS
Preterm labor, PROM, cytokines, interleukin

During the last several years a body of evidence has accumulated suggesting that preterm labor (PTL) and premature rupture of the fetal membranes (PROM) are causally related to subclinical infection. The findings of such studies supportive of this premise include the isolation of microorganisms from amniotic fluid (AF) in 15% of preterm labor pregnancies[1] and the presence of bacterial endotoxin in amniotic fluid in approximately 30% of preterm labor pregnancies.[2,3] Additionally, the inflammatory microbial products associated with subclinical infection, such as cytokines, are elevated in amniotic fluid of preterm pregnancies. For example, interleukin (IL)-1β is found in AF of approximately 50% of PTL pregnancies[4]; second, high levels of IL-6 are present in AF of approximately 50% of PTL pregnancies[5]; third, increased concentrations of IL-8 are found in AF of PTL pregnancies associated with intraamniotic infection (IAI)[6]; fourth, tumor necrosis factor (TNF)-α is present in AF of some PTL pregnancies[7,8]; and, finally, prostaglandins are identified in AF of infection-associated preterm labor.[9,10]

Analysis of fetal behavior has recently been used to evaluate patients with PROM. The findings of several studies suggest that the infected fetus behaves differently from the noninfected fetus.[11–14] In particular, a decrease in the amount of fetal

**Address correspondence/reprint requests to Dr. Susan M. Cox, at her present address, of University of Texas Southwestern Medical Center, Dept. of OB/GYN, 5323 Harry Hines Boulevard, Dallas, TX 75235-9032.**

Received January 19, 1993
Accepted May 11, 1993

**TABLE 1. Selected biophysical parameters of patients with preterm premature rupture of membranes (PPROM) and preterm labor (PTL) with intact fetal membranes according to presence or absence of intraamniotic infection[a]**

| | PPROM (n = 30) | | PTL (n = 69) | |
|---|---|---|---|---|
| | Infected (n = 6) | Noninfected (n = 24) | Infected (n = 8) | Noninfected (n = 61) |
| Absent fetal breathing | 1 (17) | 5 (21) | 2 (25) | 7 (11) |
| Absent fetal movement | 1 (17) | 0 | 1 (13) | 0 |
| Nonreactivity | 1 (17) | 2 (8) | 1 (13) | 5 (8) |

[a] No significant differences detected. Number, percent in parentheses.

breathing and fetal movement, as well as a nonreactive nonstress test (NST), has been observed in the presence of infection.

The purpose of this study was to determine prospectively the incidence of subclinical IAI in pregnancies complicated by PTL and PROM. Additionally, we sought to determine if absent fetal breathing, fetal body movement, or nonreactivity on admission to the labor hall could serve as an indicator of subclinical infection.

### SUBJECTS AND METHODS

The University of Kentucky (UK) Chandler Medical Center is a Level III regional referral center serving eastern and central Kentucky. The majority of patients entering this study were transferred to UK from nearby facilities, and all patients received magnesium sulfate tocolysis for transfer.

Entry criteria for this prospective study included: 1) either documented PTL (cervix $\geq$ 2 cm and 80% effaced or documented cervical change) or documented PPROM (nitrazine, pooling, and ferning tests); 2) singleton of fewer than 34 completed weeks of gestation; and 3) no other medical or obstetric complications. All patients were admitted to the labor suite for evaluation. After the patient had given informed consent, amniotic fluid was retrieved by transabdominal amniocentesis. The fluid was placed on ice and transported to the laboratory. After centrifugation at 300*g* for 10 minutes at 4°C, the supernatant was stored in polypropylene tubes at −70°C until cytokine analysis. A small amount of fluid was stored at −20°C in endotoxin free tubes until the *Limulus* amoebocyte lysate (LAL) test was performed for endotoxin detection. Amniotic fluid for microbiologic analysis was transported to the hospital laboratory in a capped plastic syringe and plated immediately for aerobic and anaerobic culture. Intraamniotic infection was defined as the presence of bacteria or endotoxin in the amniotic fluid.

The biophysical profile score was obtained in all patients by means of a real time ultrasonographic machine equipped with a 3.5 MHz curvilinear-array transducer. Scanning consisted of a 30 minute observation period and a biophysical profile score assigned as per the criteria of Manning and coworkers.[15] An NST was considered reactive if 2 episodes of fetal heart rate acceleration greater than 15 beats for 15 seconds were observed in 20 minutes. An NST was considered nonreactive if there were no accelerations in 40 minutes.

Amniotic fluid was analyzed for endotoxin as previously described by Cox and colleagues.[2] Quantification of cytokines in the AF was measured by sensitive and specific enzyme-linked immunosorbent assays (ELISA) for IL-1β (Cistron Biotechnologies, Pinebrook, NJ), IL-6 (Genzyme Corporation, Cambridge, MA), and IL-8 (R and D Systems, Minneapolis, MN). These assays were validated for use in AF by determining nonspecific binding and parallelism of the assay.

Analysis of categorical data was performed using 2 × 2 contingency tables with the two-tail Fisher's exact test. Continuous data were analyzed using the Mann-Whitney two-sample statistic. A *P* value less than 0.05 was considered significant.

### RESULTS

The outcome of the patients in the study is summarized in Table 1. Amniotic fluid was retrieved from all 99 patients. No patients met the criteria for clinical chorioamnionitis, such as the presence of fever, maternal tachycardia, fetal tachycardia, and uterine tenderness. Of the 30 patients with PPROM on admission, 20% had laboratory signs

TABLE 2. Amniotic fluid analysis and outcomes in patients with premature rupture of the fetal membranes[a]

| | IAI (+) (n = 6)[b] | IAI (−) (n = 24)[b] | P |
|---|---|---|---|
| Culture positive [no. (%)] | 3 (50) | 0 | 0.005 |
| LPS positive [no. (%)] | 4 (67) | 0 | <.001 |
| Mean IL-1β (ng/mL) | 18.6 ± 7.1 | — | 0.027 |
| Mean IL-6 (ng/mL) | 10.6 ± 2.4 | 0.26 ± 0.01 | 0.008 |
| Mean IL-8 (ng/mL) | 90.1 ± 1.7 | 1.6 ± 0.06 | 0.004 |
| Fetal breathing absent [no. (%)] | 1 (17) | 4 (16) | NS |
| Fetal movement absent [no. (%)] | 1 (17) | 0 | NS |
| Nonreactivity [no. (%)] | 1 (17) | 2 (8) | NS |
| Discharged undelivered [no. (%)] | 0 | 3 (13) | NS |
| Mean time to delivery (d) | 5.5 ± 1.0 | 6.9 ± 1.8 | NS |

[a] IL, interleukin; LPS, lipopolysaccharide.
[b] IAI (+), intraamniotic infection present; IAI (−), IAI absent; —, not detectable; NS, not significant.

TABLE 3. Amniotic fluid analysis and outcomes in pregnancies complicated by preterm labor (intact membranes)[a]

| | IAI (+) (n = 8)[b] | IAI (−) (n = 61)[b] | P |
|---|---|---|---|
| Culture positive [no. (%)] | 3 (38) | 0 | 0.001 |
| LPS positive [no. (%)] | 5 (62) | 0 | <0.001 |
| Mean IL-1β (ng/mL) | 3.2 ± 0.5 | — | <0.001 |
| Mean IL-6 (ng/mL) | 9.0 ± 1.6 | 0.3 | <0.001 |
| Mean IL-8 (ng/mL) | 89.8 ± 13.3 | 1.9 ± 0.17 | <0.006 |
| Fetal breathing absent [no. (%)] | 1 (13) | 11 (18) | NS |
| Fetal movement absent [no. (%)] | 0 | 0 | — |
| Nonreactivity [no. (%)] | 1 (13) | 4 (7) | NS |
| Discharged undelivered [no. (%)] | 1 (13) | 45 (74) | 0.001 |

[a] IL, interleukin; LPS, lipopolysaccharide.
[b] IAI (+), intraamniotic infection present; IAI (−), IAI absent; —, not detectable.

of infection (6 of 30 patients), while only 11.6% of the PTL group had signs of infection (8 of 69 patients). The biophysical profile was similar in the PROM and PTL infected groups (Table 1).

The outcome of the PROM study patients is summarized in Table 2. Three of the 30 patients (10%) had a positive AF culture, and 2 of these patients were also lipopolysaccharide (LPS) positive. Of the culture-negative AF(s), two were LPS-positive. An additional patient who was both culture-negative and LPS-negative had high levels of cytokines (i.e., IL-1β, IL-6, and IL-8) and therefore was considered in the IAI group. Of the six patients who had IAI, the mean levels of inflammatory cytokines, i.e., IL-1β, IL-6, and IL-8, were 18.6 ng/mL (range 1.1 to 97.8 ng/mL), 10.6 ng/mL (range 1.0 to 33.2 ng/mL), and 90.1 ng/mL (range 1.1 to 230.8 ng/mL), respectively. One of the pregnancies complicated by infection had absent fetal breathing, one had absent fetal movement, and one had no reactivity on a nonstress test. On the other hand, the group with no evidence of intraamniotic infection had AF levels of inflammatory cytokines that were undetectable (IL-1β) or at the lower limits of assay sensitivity (IL-6 and IL-8).[1] In patients with no evidence of IAI, however, four of the fetuses lacked fetal breathing movements, and two had nonreactive nonstress tests (Table 3). The mean time to delivery in the infected vs. noninfected group was 5.5 and 6.9 days, respectively (range 0.75 to 14 days in infected group vs. <1 to >30 days in the noninfected group).

The outcome of the preterm labor pregnancies with intact fetal membranes is summarized in Table 3. Eight of the 69 patients (11.6%) had evidence of IAI: 3 of these patients were culture-positive, and an additional 5 patients who were culture negative had endotoxin in the amniotic

**TABLE 4. Results in those pregnancies that delivered within 48 hours of amniocentesis and biophysical profile[a]**

| | PPROM (n = 10) | | | Preterm labor (n = 17) | | |
|---|---|---|---|---|---|---|
| | IAI (+) (2) | IAI (−) (8) | *P* | IAI (+) (4) | IAI (−) (13) | *P* |
| Culture positive (no.) | 1 | 0 | NS | 1 | 0 | NS |
| LPS positive (no.) | 1 | 0 | NS | 3 | 0 | 0.006 |
| IL-1β (ng/mL) | 5.6 ± 4.6 | — | 0.037 | 4.8 ± 1.2 | — | 0.003 |
| IL-6 (ng/mL) | 23.7 ± 9.4 | — | 0.037 | 13.3 ± 4.4 | 0.42 | 0.003 |
| IL-8 (ng/mL) | 148.2 ± 82.6 | 0.96 | 0.037 | 154.7 ± 28.2 | 5.2 ± 1.0 | 0.005 |
| Fetal breathing absent [no. (%)] | 0 | 2 (25) | NS | 0 | 3 (23) | NS |
| Fetal movement absent [no. (%)] | 1 (50) | 0 | NS | 0 | 0 | NS |
| Nonreactivity [no. (%)] | 1 (50) | 0 | NS | 1 (25) | 2 (15) | NS |

[a]IAI (+), intraamniotic infection present; IAI (−), IAI absent; IL, interleukin; LPS, lipopolysaccharide; PPROM, preterm premature rupture of membranes; —, not detectable; NS, not significant.

fluid. In the infection-positive group, the mean levels of IL-1β and IL-6 were 3.2 ng/ml (range 0.1 to 8.9 ng/mL) and 9.0 ng/mL (range .07 to 34.7 ng/mL), respectively. The mean level of IL-8 in the infected group was 89.8 ng/mL (range 0.4 to 240.4 ng/mL). In the noninfected group, IL-1β was not detected in the AF samples analyzed. With respect to IL-6, the majority of AF samples (75.4%) had no IL-6 detected; however, there was a subset of patients (n = 15) in whom low levels of IL-6 were identified (range 0.23 to 1.5 ng/mL). In two patients of this subset, cervical cultures were positive for group B streptococcus, and the intraamniotic IL-6 was 1.5 ng/mL in both samples. In patients who were negative for cervical group B streptococcus (n = 13), IL-6 was 0.2 ng/mL to 0.9 ng/mL. Fetal breathing was absent in 1 patient of the infected group and in 11 study patients of the group without infection, while fetal movement was present in all patients (Table 3). Reactivity was absent in one patient of the infected group and in four patients of the group without infection.

The time from obtaining amniotic fluid and biophysical profile was greater than 2 days in the majority of patients; therefore, we independently analyzed the group of patients who delivered within 48 hours of sampling (Table 4). In the PPROM group, two patients had evidence of infection, with one patient culture-positive and another with LPS present. Cytokines were present in both patients with evidence of infection (mean level IL-1β = 5.6 ng/mL; IL-6 = 23.7 ng/mL; and IL-8 = 148.2 ng/mL). In only one patient was fetal movement absent, and this pregnancy was in the infected group. Two fetuses had absent fetal breathing and movement, and both were in the noninfected group. One fetus was nonreactive (in the infected group).

In the pregnancies delivered within 48 hours and complicated by preterm labor, four patients (23.5%) had infection (Table 4). The mean levels of cytokines in these four patients were: IL-1β = 4.8 ng/mL; IL-6 = 13.3 ng/mL; and IL-8 = 154.7 ng/mL. Fetal breathing was absent in three study patients, all from the noninfected group. Fetal movement was present in all patients in both groups. Nonreactivity was identified in one patient of the infected group and in two patients of the group without infection.

### DISCUSSION

Current management strategies for preterm premature rupture of fetal membranes employ evaluation of fetal behavioral state. A low biophysical profile score is correlated with clinical chorioamnionitis,[16] but the correlation with subclinical infection is less well studied. Roussis and colleagues in 1991[14] reported that absent fetal breathing movements and a nonreactive nonstress test should serve as indicators for further testing (i.e., amniocentesis) to exclude infection. Other investigators have also examined the relationship between infection and lack of fetal activity. The conclusions from these studies support the premise that decreased fetal activity is accurate in predicting neonatal sepsis and/or infection.[11,13]

Recently, a growing body of evidence suggests that inflammatory markers may be a more sensitive index of intraamniotic infection than bacteriologic studies. To document subclinical IAI, we quantitated several of the inflammatory markers (i.e.,

IL-1β, IL-6, and IL-8) in AF. The results of our cytokine data are similar to the results obtained in Dr. Romero's laboratory. We both found high levels of IL-1β and IL-6 in AF from pregnancies with intraamniotic infection.[4,5] With respect to IL-8, Dr. Romero observed mean AF levels of 120 ng/ml in preterm labor pregnancies complicated by infection. The AF of women with PTL and no evidence of infection contained higher levels of IL-8 if they did not respond to tocolysis and they delivered than those not in labor (7 ng/mL vs. undetectable). These levels of IL-8 are very similar to the levels of IL-8 found in the present study (preterm delivery with infection = 154.7 ng/mL; preterm delivery without infection = 5.2 ng/mL; and PTL with no evidence of infection = 1.9 ng/mL).

In the present study subclinical IAI was not associated with a low biophysical profile score. We found no correlation between subclinical infection and a lack of reactivity, absent fetal breathing, or absent fetal movement. Thus alterations in the biophysical profile may not be an accurate predictor of subclinical IAI. However, it is possible that with the small sample size (only 14 patients had biomolecular evidence of subclinical infection), our failure to identify such a relationship may represent a type II error. If the prevalence of abnormal tests (absent fetal breathing, absent fetal tone, and nonreactivity) is 0.25 in the noninfected and 0.75 in the infected group, then inadequate power was present in this study to detect such differences (power = 0.67, 0.18 for total and <48 hours in preterm labor group; power = 0.41, 0.03 for total and <48 hours in preterm PROM group). Further, the validity of ascribing predictive value in the total sample when not stratifying for time to delivery is questionable. Developing infection cannot be determined from static lipopolysaccharide values and positive cultures obtained by amniocentesis on admission if the infection is subsequently initiated. Still, six of these pregnancies delivered within 48 hours of evaluation, and there was still no association with low biophysical parameters (i.e., absent fetal breathing and movement). Likewise, of the 85 patients without IAI, none were culture-positive, none had LPS present, and none of the patients had elevated levels of cytokines, although the biophysical profile was altered in up to 20% of these patients (Table 3, fetal breathing absent).

From these data we could not conclude that fetal biophysical parameters correlate with subclinical intraamniotic infection. Oligohydramnios limits the general usefulness of amniocentesis and subsequent analysis of biomolecular markers of inflammation. At the present time, there is no ideal test available to diagnose infection in PPROM and PTL. Obviously, further studies are needed in this critical area.

### ACKNOWLEDGMENTS

The authors are indebted to Mr. Kevin M. Knox and Ms. Pamela S. Conway for their technical assistance. The help of Mr. Sam Keeble and Ms. Carole Moore during isolation, preparation, and storage of the amniotic fluid samples is gratefully acknowledged. The authors are appreciative of the assistance of Ms. Darleen Chamberlain in the preparation of the manuscript. This work was sponsored by a grant from the NIH, 1-KO8-HDO0853.

### REFERENCES

1. Cox SM: Infection-induced preterm labor. In Glistrap LC, III, Faro S (eds): Infections in Pregnancy. pp 247–253, 1990.
2. Cox SM, MacDonald PC, Casey ML: Assay of bacterial endotoxin lipopolysaccharide in human amniotic fluid: Potential usefulness in diagnosis and management of preterm labor. Am J Obstet Gynecol 159:99–106, 1988.
3. Romero R, Rolanksy P, Oyarzun E, et al.: Labor and infection: Bacterial endotoxin in amniotic fluid and its relationship to the onset of preterm labor. Am J Obstet Gynecol 158:1044–1049, 1988.
4. Romero R, Brody DT, Oyarzun E, et al.: Infection and labor; Interleukin-1: A signal for the onset of parturition. Am J Obstet Gynecol 160:1117–1123, 1989.
5. Romero R, Avila C, Santhanam U, Sehgal PB: Amniotic fluid interleukin 6 in preterm labor. J Clin Invest 85: 1392–1400, 1990.
6. Romero R, Ceska M, Avila C, Mazor M, Behnke E, Lindley I: Neutrophil attactant/activating peptide-1/interleukin-8 in term and preterm parturition. Am J Obstet Gynecol 165:813–820, 1991.
7. Casey ML, Cox SM, Beutler B, Milewich L, MacDonald PC: Cachectin/tumor necrosis factor-α formation in human decidua. J Clin Invest 83:430–436, 1989.
8. Romero R, Manogue KR, Mitchell MD, et al.: Infection and labor: Cachectin-tumor necrosis factor in the amniotic fluid of women with intraamniotic infection and preterm labor. Am J Obstet Gynecol 161:336–441, 1989.
9. Romero R, Emamian M, Wan M, et al.; Prostaglandin concentrations in amniotic fluid of women with intraamniotic infection and preterm labor. Am J Obstet Gynecol 157:1461–1467, 1987.

10. Casey ML, Cox SM, Word RA, MacDonald PC: Cytokines and infection-induced preterm labour. Reprod Fertil Dev 2:499–509, 1990.
11. Vintzileos AM, Campbell WA, Nochimson DJ, Weinbaum PJ: Fetal breathing as predictors of intraamniotic infection in premature rupture of membranes. Obstet Gynecol 57:813–817, 1986.
12. Goldstein J, Romero R, Merrill S, et al.: Fetal body and breathing movements as predictors of intraamniotic infection in preterm premature rupture of membranes. Am J Obstet Gynecol 159:363–368, 1988.
13. Vintzileos AM, Winston WA, Nochimson DJ, Connolly ME, Fuenfer MM, Hoehm GJ: The fetal biophysical profile in patients with premature rupture of the membranes: An early predictor of fetal infection. Am J Obstet Gynecol 152:510–516, 1985.
14. Roussis P, Rosemond RL, Glass C, Boehm FH: Preterm premature rupture of membranes: Detection of infection. Am J Obstet Gynecol 165: 1099–1104, 1991.
15. Manning FA, Baskett TF, Morrison I, Lange I: Fetal biophysical profile scoring: A prospective study in 1184 high risk pregnancies. Am J Obstet Gynecol 140:289–294, 1981.
16. Ohlsson A, Wang E: An analysis of antenatal tests to detect infection in preterm premature rupture of the membranes. Am J Obstet Gynecol 162:809–818, 1990.
